# Molecular Mechanisms of Mitochondrial Quality Control in Ischemic Cardiomyopathy

**DOI:** 10.7150/ijbs.76223

**Published:** 2023-01-01

**Authors:** Xing Chang, Ruxiu Liu, Ruibing Li, Youyou Peng, Pingjun Zhu, Hao Zhou

**Affiliations:** 1 Guang'anmen Hospital of China Academy of Chinese Medical Sciences, Beijing, China.; 2 Department of Clinical Laboratory Medicine, The First Medical Centre, Medical School of Chinese People's Liberation Army, Beijing, China.; 3 Senior Department of Cardiology, The Sixth Medical Centre of People's Liberation Army General Hospital, Beijing, China.; 4 Department of Respiratory and Critical Care Medicine, The Second Medical Center & National Clinical Research Center for Geriatric Diseases, Chinese PLA General Hospital, Beijing, China.; 5 Montverde Future Academy Shanghai, 88 Jianhao Road, Pudong New District, Shanghai, China.

**Keywords:** Ischemic cardiomyopathy, mitochondrial quality control, mitophagy, mitochondrial dynamics, mitochondrial biogenesis, calcium signal

## Abstract

Ischemic cardiomyopathy (ICM) is a special type of coronary heart disease or an advanced stage of the disease, which is related to the pathological mechanism of primary dilated cardiomyopathy. Ischemic cardiomyopathy mainly occurs in the long-term myocardial ischemia, resulting in diffuse myocardial fibrosis. This in turn affects the cardiac ejection function, resulting in a significant impact on myocardial systolic and diastolic function, resulting in a decrease in the cardiac ejection fraction. The pathogenesis of ICM is closely related to coronary heart disease. Mainly due to coronary atherosclerosis caused by coronary stenosis or vascular occlusion, causing vascular inflammatory lesions and thrombosis. As the disease progresses, it leads to long-term myocardial ischemia and eventually ICM. The pathological mechanism is mainly related to the mechanisms of inflammation, myocardial hypertrophy, fibrosis and vascular remodeling. Mitochondria are organelles with a double-membrane structure, so the composition of the mitochondrial outer compartment is basically similar to that of the cytoplasm. When ischemia-reperfusion induces a large influx of calcium into the cell, the concentration of calcium ions in the mitochondrial outer compartment also increases. The subsequent opening of the membrane permeability transition pore in the inner mitochondrial membrane and the resulting calcium overload induces the homeostasis of cardiomyocytes and activates the mitochondrial pathway of apoptosis. Mitochondrial Quality Control (MQC), as an important mechanism for regulating mitochondrial function in cardiomyocytes, affects the morphological structure/function and lifespan of mitochondria. In this review, we discuss the role of MQC (including mitophagy, mitochondrial dynamics, and mitochondrial biosynthesis) in the pathogenesis of ICM and provide important evidence for targeting MQC for ICM.

## Introduction

**Figure 1:**
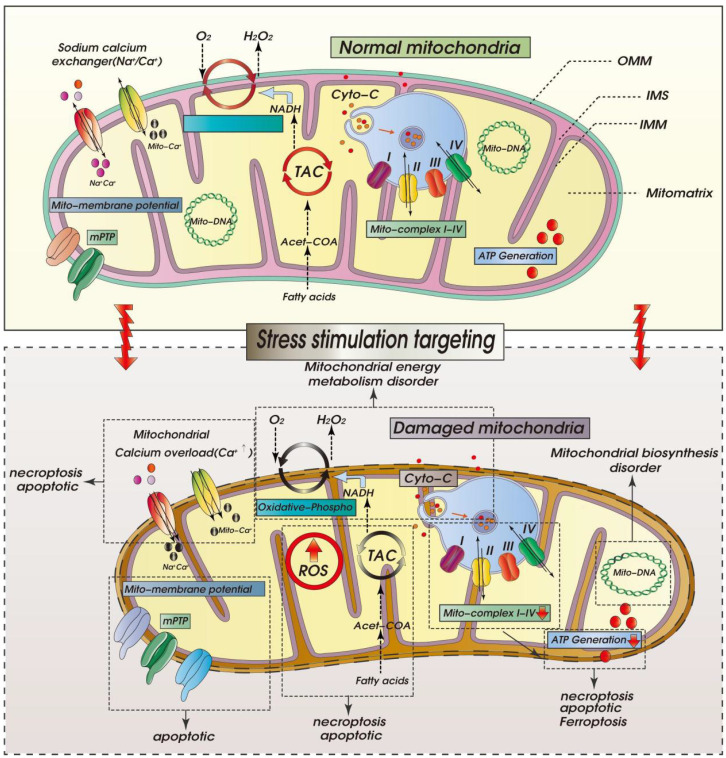
**ROS-mediated mitochondrial dysfunction in ICM. Legend:** Oxidative stress damage caused by excessive accumulation of ROS can seriously damage mitochondrial function. When mitochondria are dysfunctional, mitochondrial inner and outer membrane permeability (OMM) functions are also dysfunctional, which leads to abnormal opening of mitochondrial permeability transition pore (mPTP). It is also accompanied by calcium homeostasis disorder and tricarboxylic acid circulation disorder. Calcium signal conduction dysfunction and mitochondrial energy metabolism (mitochondrial respiration) dysfunction also occur.

ICM is a special type or late stage of coronary heart disease. It is mainly due to the long-term ischemia/hypoxia caused most commonly by coronary atherosclerosis, which leads to myocardial hypertrophy or stiffness, congestive heart failure and/or arrhythmia 1-3. Other conditions that can lead to ICM include acute myocardial infarction accompanied by coronary microvascular damage or endothelial damage, coronary artery spasm, embolism, congenital coronary artery malformation and coronary arteritis 4. The heart is different from other organs. When oxygen consumption by the heart increases, it can only meet that demand through coronary blood flow. Consequently, coronary stenosis that causes decreases regional blood flow will often lead to myocardial ischemia 5-7. The main causes of coronary stenosis and myocardial ischemia are coronary atherosclerosis, arterial thrombosis, vascular inflammation/injury and coronary microvascular injury/abnormal coronary artery structure 8, 9. The clinical symptoms of ICM are angina pectoris, heart failure, arrhythmia, thrombosis/embolism, dyspnea and so on (Fig. 1). Some patients also exhibit pulmonary edema. In late stage of ICM, heart failure, serious arrhythmia, and even death will occur 10. Numerous studies have shown that the development of the pathological mechanism of ICM is closely related to mitochondrial quality control (MQC). The MQC includes mechanisms such as mitophagy, mitochondrial biosynthesis, mitochondrial fusion and fission. MQC can maintain a certain level of mitochondrial homeostasis and mitochondrial energy metabolism, thereby maintaining the homeostasis of the intracellular environment and the physiological function of cardiomyocytes. In this review, we discuss the role of MQC in different pathological mechanisms of ICM, and provide references for targeted therapy of the complex mechanisms of ICM.

## Molecular Pathophysiology of ICM

### Inflammation

The pathogenesis of ICM involves coronary artery stenosis caused by atherosclerosis, which affects the supply of blood oxygen, but its essence is inflammatory 11. This inflammatory process underlying ICM involves the participation of a variety of pathological mechanisms and is a stress response to myocardial tissue injury or coronary microvascular endothelial injury. It is also an inevitable mechanism contributing to the healing process after myocardial injury 12. In the late stage of acute myocardial infarction, there will be a long-lasting inflammatory reaction within the myocardium or vascular endothelium, which can lead to the rupture of coronary atherosclerotic plaque and cause more serious damage. Neutrophils, lymphocytes, and monocyte macrophages in the blood circulation will be activated and release interleukin-6 (IL-6). This rapid increase of inflammatory markers and inflammatory cells in the circulation after myocardial infarction is consistent with the increase of the recurrence rate of cardiovascular events and is a predictor of recurrent cardiovascular events 13.

The explosive inflammatory response can cause great injury to myocardium 14. Common cardiovascular risk factors such as high sugar, high fat, high salt diet, smoking, hypertension and insulin resistance can lead to chronic inflammation, redox imbalance and mitochondrial homeostasis disorder 15. In addition, oxidized or modified LDL will attract white blood cells into the coronary intima, which are phagocytosed by macrophages, resulting in the formation of foam cells further development of atherosclerotic plaques. The growth of foam cells will gradually increase the coronary arterial occlusion, leading to a mismatch between myocardial oxygen demand and oxygen supply, which can result in myocardial infarction or myocardial ischemic injury 16. Moreover, the inflammatory reaction can be spread by modified LDL, leading to coronary endothelial or microvascular injury, fiber proliferation reaction and thrombosis in subintimal arteries, which can exacerbate acute coronary artery stenosis and myocardial ischemic injury 17. Notably, within tissues under the stress of coronary ischemia, the inflammatory response also plays a transient protective role. The inflammatory response indirectly maintains the integrity of cardiac structure and cardiac function. However, if the inflammation persists and is not effectively controlled, it will lead to ICM.

Cardiomyocyte necrosis and apoptosis is accompanied by inflammatory reaction. This pro-inflammatory stage aims to remove damaged cardiomyocytes or vascular endothelial cells. However, in the process of removing damaged cells and promoting wound healing may lead to excessive activity of fibroblasts and replacement of cardiomyocytes with active fibroblasts, resulting in scar formation or myocardial fibrosis, which is also one of the reasons for serious myocardial fibrosis and myocardial hypertrophy are seen in later stages of ICM 18, 19. In coronary artery endothelial cells, pro-inflammatory cytokines also induce the expression of adhesion molecules and promote immune cell adhesion to endothelium and transendothelial migration. It can also induce explosive production of reactive oxygen species (ROS), accelerate the process of endothelial cell apoptosis, stimulate the formation of collagen in microvascular endothelial cells and dysfunction of the fibrinolytic system 20. It is therefore an important pathological basis for microvascular injury, the no reflow phenomenon and ICM in the later stage of myocardial ischemia and in acute myocardial infarction 18.

The inflammatory mechanism regulating NLRP3 is closely related to mitochondria, which play a key role in the initiation and regulation of NLRP3 inflammatory bodies or inflammasomes. NLRP3 activation can further induce mitochondrial homeostasis imbalance, induce NLRP3 de‑ubiquitination and the release of mitochondrially derived molecules and destroy mitochondrial DNA 21. When mitophagy is blocked, mitochondrial ROS will have a cumulative effect that acts indirectly to activate NLRP3. Static NLRP3 is located within the endoplasmic reticulum. However, when inflammatory bodies are activated, NLRP3 is redistributed to the space around the nucleus and colocalizes with mitochondria, resulting in the destruction of mitochondrial structure and respiratory chain function, which further enhances production of ROS 22, 23.

It appears that mitochondria not only regulate NLRP3 activation, but are also the docking site for inflammatory body assembly. In stress stimulated cells, NLRP3 is activated and associated with mitochondria and mitochondrial associated membranes (MAMs) 24. Mitochondria may also act as an “adapter” for NLRP3 activation, and the ROS production and redox imbalance associated with mitochondrial damage are important drivers of NLRP3 inflammatory body activation.

Current evidence suggests that mechanism regulating inflammatory is an important pathological factor contributing to ICM, and ROS bursts and NLRP3 activation mediated by stress-induced mitochondrial dysfunction may be key factors inducing ICM inflammation. Mitochondria may act as a “Trojan horse” to induce inflammatory storms, but they have dual regulatory roles, as can maintain the basic function of cardiomyocytes in the context of oxidative stress and inflammation 23.

### Oxidative stress

Oxidative stress is defined as tissue destruction caused by an imbalance between ROS production and cell redox, which will have a variety of negative effects on the metabolic function and physiological activities of cardiomyocytes 25. It is also an important regulatory mechanism that induces apoptosis and other forms of programmed cell death and is a key factor contributing to myocardial and vascular endothelial injury in ICM.

ROS are short-lived, low-molecular weight compounds, mainly derived from various redox reactions. Increases in ROS can alter cell signaling proteins and produce corresponding functional consequences that mediate the pathophysiological process of ICM 26. ROS are mainly produced in the mitochondria. In healthy myocardium, ROS are an accidental by‑product of mitochondrial respiration, and their levels are controlled by antioxidant enzymes. Under many pathological conditions, it is difficult to maintain redox balance, and when the antioxidant cell defense system is inhibited by oxidative stress, mitochondrial dysfunction, apoptosis or other forms of programmed cell death can occur. Within the myocardium, excessive accumulation of ROS lead disruption of calcium homeostasis, cardiac remodeling induced by transforming growth factor signal transduction, apoptosis and necrosis 27. In the pathogenesis of ICM, ROS production also interacts with inflammatory processes, fibrosis and dysregulation of mitochondrial homeostasis and has negative effects on angiogenesis, vascular tone and endothelial cell genome stability.

Under normal physiological conditions, ROS are rarely produced in the heart. Superoxide leaking from the mitochondrial electron transport chain is quickly reduced to water and molecular oxygen by the antioxidant enzyme system. The heart can cope with redox changes and shows a significant adaptive response to maintain normal contractility. Mitochondria are the “hub” of cell redox process. Many mitochondrial stress signals participate in intercellular signal transduction and interaction mechanisms to regulate adaptation of cardiomyocytes to exogenous and endogenous stimuli. Oxidative stress is sensed by mitochondria, which initiate the release of stress signals that regulate membrane polarization, adenine nucleotide levels, ROS production, excitation/contraction coupling and opening/closing of membrane permeability transition pores 28. These mechanisms are key determinants of the recovery response of cardiomyocytes after ischemic injury.

As shown in Figure 1, within ischemic cardiomyocytes, a rapid increase of ROS induces electron leakage from mitochondrial respiratory complexes I and III, which disrupts release of cardiolipin. Resultant changes in the phospholipid composition of the inner mitochondrial membrane will lead to the instability of the electron transport chain complex and super-complex, ultimately leading to mitochondrial structural disruption and dysfunction 29. As a result, cell energy metabolism (ATP production) will be diminished, leading to additional ROS production and completion of a vicious cycle. When the compensatory response of cardiomyocytes is continuously stimulated, there is excessive proliferation and hypertrophy of fibroblasts, which leads to development of myocardial fibrosis and cardiac dysfunction 30. In hearts affected by ICM, chronic aseptic inflammation caused by myocardial ischemia, excessive accumulation of ROS will mediate the release of inflammatory cytokines and activate matrix metalloproteinases, resulting in collagen degradation. This may lead to myofibril disorder and ventricular remodeling and dilation. The ROS production stimulated by inflammatory cells also disrupts cellular ion exchange by inhibiting the activities of sarco/endoplasmic reticulum Ca^2+^-ATPase (SERCA2a) and sarcolemmal (Na^+^ K^+^) Ca^2+^-ATPase, which results in decreased Ca^2+^ efflux and increased Ca^2+^ influx, ultimately leading to intracellular Ca^2+^ overload and cell death 31.

ROS mediated mitochondrial dysfunction is particularly damaging to the coronary vascular system, as the amount of oxygen delivered to the myocardium is greater than to other tissues or organs. The development of ICM increases the duration of myocardial ischemia and can lead to necrosis. Although early reperfusion can restore blood flow to the affected area, the myocardial injury caused by reperfusion injury may be much higher than that caused by ischemia itself. This is in large part because reperfusion may induce the explosive ROS production, and the resultant oxidative stress injury to the myocardium may be more serious than the ischemic injury 32. Targeting the regulation of mitochondrial function or energy metabolism may be an important target for relief of oxidant stress in ICM, and this intervention should run through all stages of ICM.

### Cell death

The cell death seen in patients with ICM has significant prognostic value with respect to cardiac function, quality of life and survival after coronary revascularization. Although numerous animal studies and clinical investigations have been performed to explore the appropriate detection modalities for cardiomyocyte death (e.g., dobutamine stress echocardiography 33, late contrast enhancement of magnetic resonance imaging 34, and technetium-99m single positron emission computed tomography 35), it remains a mystery as to where cardiomyocyte death occurs exactly and how cardiomyocyte death is induced in different stages of ICM.

Apoptosis is a form of programmed cell death that contributes significantly to cardiomyocyte loss during ICM. It is characterized by sequential activation of caspase family enzymes, which is reportedly the downstream signaling cascade induced by mitochondria damage, endoplasmic reticulum (ER) stress or extracellular signals. Mitochondrial dysfunction promotes the leakage of mitochondria-localized pro-apoptotic factors (i.e., Cyt-C, Smac and HtrA2/Omi) into cytosol stimulating activation of caspase-3/-9. ER stress is an adaptive response to attenuate at the transcription level the buildup of abnormal proteins within the ER. However, uncontrolled ER stress augments the expression of caspase-12, which contributes to caspase-3 activation. In addition to organelle-mediated apoptosis, extracellularly stimulated apoptosis is initiated via the death receptor 36. In brief, the binding of an apoptotic ligand (e.g., FasL or TNF-α) to its death receptor promotes the formation of death-inducing signaling complex (DISC).

Necrosis accounts for only about 90,000 cardiomyocyte deaths 2 h after coronary artery occlusion and has no effect on the non-ischemic myocardium. Interestingly, necroptosis, a regulated form of necrosis, reportedly accounts for more than 50% of cardiomyocyte death during acute myocardial ischemia. Necroptosis is primarily induced by the activation of intracellular serine/threonine kinases receptor interacting protein kinase 1 (RIPK1) and RIPK3 as a result of inflammatory responses or oxidative stress 37. This signal transduction pathway is accepted as the classical necroptotic cell death pathway involved in cardiomyocyte death during myocardial ischemia, myocardial infarction, and post-infarction cardiac injury. On the other hand, RIPK3 also phosphorylates PGAM5, a mitochondrial protein phosphatase participating in mitochondrial fission and mitophagy 38. Cyclophilin D (CypD), is phosphorylated by PGAM5 at Ser31, which enhances the mPTP opening rate. As a complex channel bridging the inner and outer mitochondrial membranes, the long-term opening of mPTPs leads to mitochondrial swelling and ATP exhaust. Ca^2+^-calmodulin-dependent protein kinase (CaMKII) is another phosphorylation substrate of RIPK3 37. Like CypD, phosphorylated CaMKII is capable enhancing mPTP opening through a mechanism involving ROS overload.

It appears that levels of both RIPK3 and phospho-MLKL are significantly upregulated in ischemic cardiomyocytes. Genetic or pharmacological blockade of necroptosis pathway molecules increases resistance to ischemic challenge and reduces infarct size, with a prominent improvement in cardiac function 39. In addition to evidence from animal experiments, the necroptotic ratio, as revealed by RIPK1/3 staining and TUNEL, is significantly increased in myocardial samples from patients with ICM undergoing cardiac transplantation. By contrast, control samples (from the hearts of patients who died from non-cardiac causes) showed only faint and very scarce staining of RIPK1/3 staining and TUNEL 40. Interestingly, a subsequent study reported that RIPK3 expression is primarily upregulated in the left ventricle of failing hearts 6 weeks after 60-min coronary occlusion, whereas RIPK3 expression is downregulated in right ventricle. On the other hand, MLKL expression is only elevated in the right ventricle and is unhanged in the left ventricle of failing heart 6 weeks after 60-min coronary occlusion 41. The basis of these discrepancies is still unclear but indicate different responses and/or different signals regulating necroptotic cell death in left/right ventricles during myocardial ischemia.

### Fibrosis

At later stages, ICM is accompanied by myocardial hypertrophy and heart failure, which are closely related to myocardial fibrosis. Myocardial fibrosis is an important part of tissue repair after myocardial ischemic injury. It is the result of persistent and repeatedly aggravated myocardial ischemia and hypoxia caused by coronary stenosis 42. Myocardial fibrosis is the product of multiple pathological processes, including reactive interstitial fibrosis and perivascular fibrosis. Myocardial fibrosis can disrupt intracellular energy metabolism and the redox balance, adversely affecting the nutrition supply to myocardium 43 and inducing chronic inflammation, vascular remodeling and cell death. It can also lead to the development of ICM and, eventually, heart failure. With progressive myocardial fibrosis, the resultant cardiac cavity expansion can lead to increased ventricular wall thickness with multifocal white fiber cords or patches, and even transmural scarring 44. The endocardium thickens and organic mural thrombosis is seen. Fibrotic injury of the myocardium is also accompanied by local atrophy with hypertrophy in areas adjacent to the fibrosis. Progression of the condition induces increases in heart volume and weight, which becomes the pathological basis of heart failure at later stages of ICM.

The interaction between fibroblasts and cardiomyocytes plays an important role in the course of myocardial fibrosis. Myofibroblasts can induce and regulate cardiomyocyte hypertrophy under stress or myocardial injury. Cardiac fibroblasts and cardiomyocytes interact through biochemical mediators such as TGF-β, angiotensin II (Ang-II) and various interleukins 45. Signaling between fibroblasts and cardiomyocytes also occurs through crosstalk via gap junctional proteins and biomechanical interactions. With progression of myocardial fibrosis in later stage ICM, as the thickness of the ventricular muscle and the stress on the ventricular wall increase, many cells are experience increased biomechanical strain. Mechanically sensitive adhesion proteins such as integrin and cadherin mediate mechanical signals between fibroblasts and cardiomyocytes and their microenvironment and indirectly stimulate the production of extracellular matrix (ECM) 46.

Excessive deposition of ECM within heart tissue leads to hardening of the fibrotic tissue and reduces the compliance of the myocardium. The purpose of fibrosis is to promote or accelerate deposition of connective tissue to maintain the integrity and structure of injured myocardial tissue. However, This adaptive response gradually leads to scarring of the damaged tissue 47. As cardiomyocytes are replaced by fibroblasts, myocardial tissue is disrupted and cardiac function is diminished.

Mitochondrial-derived ROS (mtROS)-induced TGF-β binds to cell surface receptors, leading to phosphorylation of the Smad transcription factor family to initiate gene expression, which may be a key factor in the proliferation and/or differentiation of fibroblasts. TGF-β-induced gene expression in fibroblasts requires mtROS produced by complex-III, and excessive accumulation of ROS can be detected in the mitochondrial matrix and cytoplasm. Application of antioxidants that target mtROS produced by complex-III reduces TGF-β-induced expression of pro-fibrotic genes 48, suggesting mtROS is an important regulator of normal TGF-β-mediated gene expression and fibrosis formation. Oxidative stress and inflammatory responses arising from mitochondrial dysfunction are the key factors contributing to myocardial fibrosis. Therapies targeting mitochondrial quality surveillance may be important targets for the treatment of myocardial fibrosis.

### Calcium signaling disorder

Dysregulation of Ca^2+^ homeostasis is closely related to the pathogenesis of ICM. Ca^2+^ overload within cardiomyocytes can be induced by inflammatory responses, oxidative stress injury and mitochondrial dysfunction, and can stimulate progression of both myocardial fibrosis and myocardial hypertrophy 49. The Ca^2+^ distribution is uneven within the myocardium. In many cell types cytosolic free Ca^2+^ acts as a second messenger transducing signals from extracellular stimuli. In cardiomyocytes at rest, the cytosolic Ca^2+^ concentration in cardiomyocytes is tightly regulated to around 10^-7^-10^-8^ mol/L through the action of SERCA2a and sarcolemmal (Na^+^ K^+^) Ca^2+^-ATPase. Transient changes in the level of cytosolic Ca^2+^ within cardiomyocytes and vascular smooth muscle mediate excitation-contraction coupling in these cells. Therefore, levels of cytosolic Ca^2+^ are strictly regulated 50. Within the ischemic myocardium, extracellular signals can increase the cytosolic Ca^2+^concentration by increasing extracellular Ca^2+^ influx through opening of Ca^2+^channels in cell membrane and Na^+^/Ca^2+^ exchange and release of Ca^2+^ from intracellular stores in the sarcoplasmic reticulum. Within the ischemic myocardium excessive influx of extracellular Ca^2+^ and release of store Ca^2+^ can lead to Ca^2+^ overload 51. Within vascular smooth muscle cells, the increased contractility caused by Ca^2+^ overload can lead to increased total peripheral blood flow resistance and hypertension, and gradually destroy the structural integrity of arteries and arteriolar walls. Vascular Ca^2+^ overload exacerbates arteriosclerosis in various animal models and coronary plaques in human, thereby contributing to the pathogenesis of ICM 52.

Mitochondria are the energy suppliers for cells. They also monitor signals from the extracellular and intracellular environment, including growth factors, ROS and DNA damage signals. In the animal models of ICM, mitochondrial dysfunction is a key factor contributing to myocardial Ca^2+^ dysregulation and injury 53. Mitochondria are also important nodes for regulation of the cytosolic free Ca^2+^ concentration by interacting with the endo/sarcoplasmic reticulum (ER/SR) through “mitochondria ER contacts.” The molecular assembly that forms this connection provides a local environment that enhances the exchange of Ca^2+^ and other signals between the two organelles. Consequently, ischemia will not only cause mitochondrial oxidative stress damage, but also induces ER/SR stress, which leads to release of Ca^2+^ from the ER/SR into the mitochondria causing mitochondrial Ca^2+^ overload 54. The resultant mitochondrial dysfunction is considered to be a “transit-station” between ER stress and cardiomyocyte Ca^2+^ overload 150.

Mitochondria are formed by a double membrane. The inner membrane contains the components involved in oxidative phosphorylation (ATP production), while the outer membrane controls mitochondrial dynamics, including their fission and fusion. In addition to helping to control levels of cytosolic Ca^2+^ and intracellular Ca^2+^ signaling, mitochondrial Ca^2+^ uptake also plays key role in regulating ATP production, autophagy, and cell death. Ca^2+^ is taken up into mitochondria primarily via mitochondrial calcium transporter. Dysregulation of mitochondrial Ca^2+^ homeostasis may trigger the opening of mPTPs in the inner membrane, which can lead to uncoupling of oxidative phosphorylation, mitochondrial swelling and mitochondrial inner or outer membrane damage 55. Under normal conditions, mPTPs allow positive ions to enter the mitochondrial matrix. When affected by ICM, however, mitochondrial permeability is changed, enabling the soluble protein apoptosis inducing factor (AIF) to be released into the cytosol, where it induces apoptosis 56.

In the pathogenesis of ICM, intracellular Ca^2+^ homeostasis is closely related to ROS-mediated oxidative stress. The redox balance within cardiac and vascular myocytes is governed by the balance between ROS production and the activities of antioxidant enzymes. Although the relationship between oxidative stress and Ca^2+^ overload associated with ICM remains to be further clarified, ROS formation under various experimental and clinical conditions may directly or indirectly disrupt intracellular calcium homeostasis 57. For instance, excessive accumulation of ROS can interfere with Ca^2+^ channels in both the SR and plasma membrane, resulting in extracellular Ca^2+^ influx and SR Ca^2+^ release. As mentioned earlier, upon reperfusion of the coronary artery and myocardium, the large influx of oxygen molecules into the ischemic tissue can also lead to the explosive production of ROS and myocardial mitochondrial damage. This will adversely affect mitochondrial oxidative phosphorylation, resulting in a lack of cell energy, which will, in turn, lead to SERCA dysfunction and release of excessive levels of Ca^2+^ from the SR, mitochondrial Ca^2+^ overload, and activation of apoptosis-related signaling 56.

In summary, the pathological mechanism underlying ICM is multifactorial and involves inflammation, oxidative stress, cell death, fibrosis and dysregulation of Ca^2+^ homeostasis (Table 1). Moreover, these mechanisms interact with each other, exacerbating the myocardial injury. Mitochondria can directly regulate intracellular Ca^2+^ homeostasis through the MCU and provide energy for cardiomyocytes through oxidative phosphorylation to promote cardiomyocyte survival. Mitochondrial can directly affect the regulatory mechanisms governing apoptosis through release of apoptotic signaling molecules that trigger the caspase apoptosis pathway. It is therefore necessary to further clarify the regulatory roles of mitochondria in the multiple mechanisms contributing to the pathogenesis of ICM.

## Mitochondrial Quality Control (MQC) in ICM

### MQC in cardiomyocyte bioenergetics and metabolism (aerobic respiration, anaerobic glycolysis)

**Figure 2:**
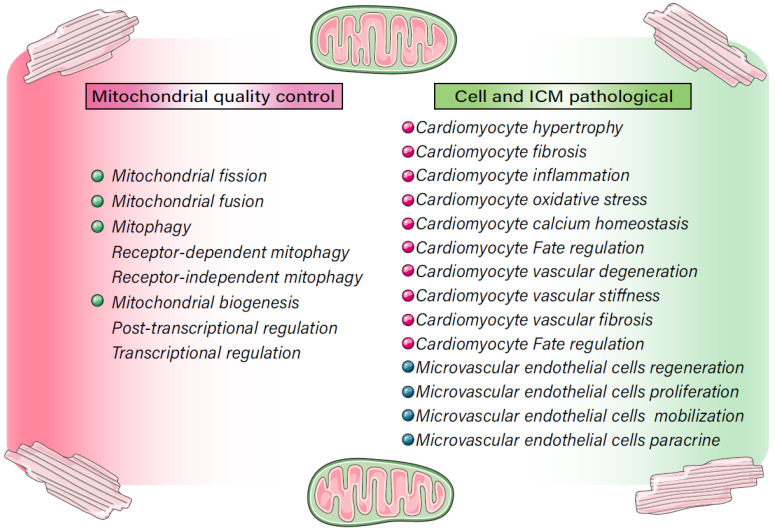
** Regulatory mechanisms involved in MQC in cell homeostasis and ICM.** MQC directly or indirectly regulates cellular internal and external stress signaling pathways, affects the activity of cardiomyocytes and microvascular endothelial cells, and contributes to multiple pathological mechanisms, including oxidative stress, cardiomyocyte hypertrophy, fibrosis, and Ca^2+^ homeostasis disorders, as well as apoptotic/necroptotic processes.

There is currently no specific treatment for ICM. The main treatment methods involve adjusting living habits and control of blood pressure, blood lipid and blood glucose. As shown in Figure 2 and Table 2, recent studies have shown MQC (including mitochondrial dynamics, mitochondrial autophagy, and mitochondrial biosynthesis) to be centrally involved in the pathological mechanism underlying ICM and have put forward new research directions and targeted treatment strategies.

Levels of active energy metabolism in the heart are the highest in the body, as the heart requires large amounts of energy to maintain systolic activity. As an adaptation to the high energy demand needed to drive the contraction cycle and maintaining ionic homeostasis, mitochondrial density within cardiomyocytes is high. Within mature cardiomyocytes, mitochondria account for one-third of the total cell volume. Moreover, they are relatively stable structures and closely related to sarcoplasmic reticulum and myofibrils, enabling them to provide energy in the form of ATP and creatine and contribute to Ca^2+^ regulation 58. About 90% of cellular ATP produced within the myocardium is used to support the systolic/diastolic cycle. Even slight changes in mitochondrial function can lead to significant changes in cardiomyocyte energy production and damage to cardiovascular health. Mitochondria are thus deeply involved in the processes ongoing with cardiomyocytes when the heart is ischemic, or cardiomyocytes are in a state of stress.

The mitochondrial energy metabolism system is composed of metabolic fuel transport and degradation pathways as well as fatty acid β-oxidation, electron transport chain, molecular motors (ATPase) and feedback signals all acting in concert in a non-equilibrium steady state. The role of mitochondrial oxidative phosphorylation in the heart is to maintain the high phosphorylation potential to replace ATP catabolized during systolic/diastolic cycling 59. Oxidative phosphorylation is an aerobic metabolic process in which electrons pass through the inner membrane of mitochondria via the electron transfer chain, ultimately reducing oxygen to water molecules. Through this reaction series, protons are pumped against their concentration gradient (ΔPHm) into the mitochondrial membrane gap 60.

The mitochondrial energy metabolism system perfectly adapts ATP synthesis to ATP hydrolysis, which is closely related to the integrity of mitochondrial morphology and structure and the normal maintenance of energy metabolism. Because the structure, function and energy metabolism level of mitochondria directly affect the metabolic processes of cardiomyocytes, MQC is an essential regulatory factor governing the survival cycle of cardiomyocytes. Myocardial energy consumption mainly depends on oxidized fatty acids, glucose, lactic acid, and ketones, which are used to produce ATP through oxidative phosphorylation. However, this is not absolute and related to the metabolic, nutritional, and stress state of the body 61. During movement, lactic acid accounts for a large proportion of the myocardial energy supply. The myocardium contains high levels of lactate dehydrogenase isozyme (LDH) 1, which catalyzes the dehydrogenation of lactic acid to, putting the reduced form of the coenzyme nicotinamide adenine dinucleotide (NADH) and pyruvate into the tricarboxylic acid (TCA) cycle to provide energy.

Mitochondria are where sugars, fatty acids and amino acids are oxidized to release the energy used for oxidative phosphorylation during ATP production. More than 95% of ATP in the mitochondria is produced through oxidation. Under normal physiological conditions, 60-90% of myocardial ATP is produced by the oxidation of fatty acids. The common pathway for final oxidation and oxidative phosphorylation is the tricarboxylic acid cycle 62. These molecules are then used to reduce oxygen and release the energy used to synthesize ATP. When the heart is in an “energy starvation state,” aerobic oxidative metabolism of fatty acids and pyruvate is significantly inhibited. The heart then depends largely on increased sugar intake and glycolysis to provide energy. When the heart in an ischemic state, glycolysis is accelerated with the increase of glucose intake, and the activity of important enzymes is maintained by providing limited amounts of ATP 63. However, when ischemia is severe, there is a decrease of glucose intake, depletion of glycogen, translocation and inactivation of phosphofructokinase, and inhibition of glyceraldehyde-3-phosphate dehydrogenase, all of which inhibit glycolysis and reduce ATP production. After ischemia/reperfusion, the recovery of fatty acid energy metabolism is faster than glucose oxidation, and the imbalance between glycolysis and glucose oxidation can further cause accumulation of metabolites (e.g., lactic acid and H^+^) exacerbating cardiomyocyte damage 64. At this time, MQC plays a key regulatory role in cell metabolism.

**Figure 3:**
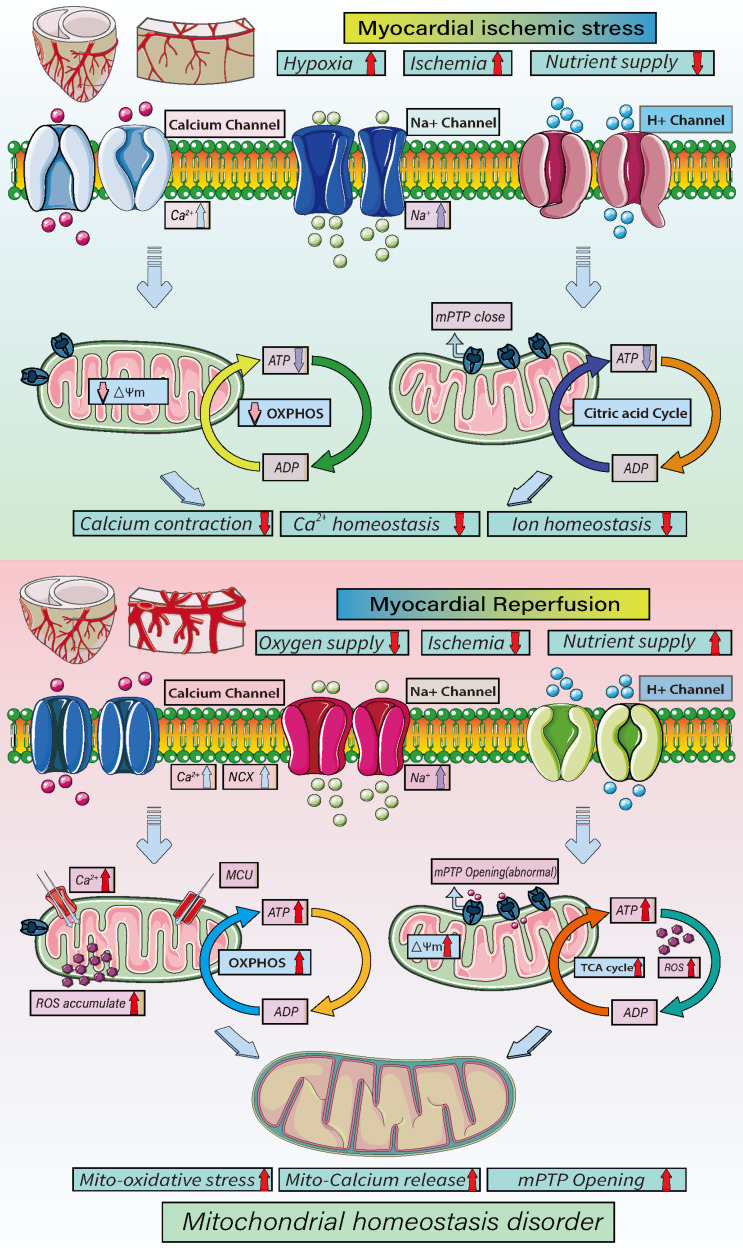
** Mechanisms underlying mitochondrial homeostasis in cellular energy metabolism.** Myocardial ischemia/reperfusion injuries contribute to the pathogenesis of ICM. During myocardial hypoxia/ischemia, and nutrients decline while Ca^2+^, Na^+^ and K^+^ channels open, lead to intracellular Ca^2+^ overload and disruption of intracellular ion homeostasis leading to a loss of mitochondrial membrane potential and decline of oxidative phosphorylation. This in turn will lead to a decline in ATP synthesis and cell energy metabolism disorders. After reperfusion, intracellular oxygen levels rise sharply, as do nutrient levels. In addition, cardiomyocytes exhibit Ca^2+^ overload and mitochondrial MCU dysfunction, exacerbation the exacerbating the Ca^2+^ overload. The transient increase in in oxygen supply leads to accumulation of ROS and increases in the mitochondrial membrane potential, which stimulates the abnormal opening of mPTPs. Oxidative phosphorylation and the tricarboxylic acid cycle are over-activated due the oversupply of blood oxygen and nutrients, leading to the overproduction of ATP and ROS, ultimately causing oxidative stress and disruption of mitochondrial homeostasis.

As shown in Figure 3, mitochondrial energy metabolism plays important regulatory roles at different stages of ICM and reperfusion injury. In acute myocardial ischemia, the oxygen supply and nutrition/energy supply are lacking, which disrupts intracellular Ca^2+^ and other ion homeostasis. At the same time, there is a loss mitochondrial energy metabolism and mitochondrial membrane potential. As a result of the decrease in oxidative phosphorylation, ATP synthesis is insufficient, and the cell death program is initiated. During reperfusion, the oxygen and nutrient supplies are recovered, but the supply exceeds the metabolic ability of cardiomyocytes, which the accumulation of mitochondrial ROS and an increase in mitochondrial membrane potential, which stimulates abnormal opening of mPTPs. In addition, the overactivation of oxidative phosphorylation and the tricarboxylic acid cycle leads to excessive ATP and ROS production and, ultimately, mitochondrial dysregulation and oxidative stress damage 61, 65.

In addition, the dysregulation of mitochondrial dynamics, mitophagy and biosynthesis caused by acute ischemia and reperfusion directly affects the cell metabolism and death processes. It has been observed, for example, that feeding mice a high-fat diet (HFD, 60 kcal% fat) activates mitophagy, which alters cardiac mitochondrial energy metabolism and fatty acid oxidation and indirectly affects the synthesis of mitochondrial DNA. However, blocking parkin-mediated mitophagy leads to mitochondrial dysfunction and lipid accumulation, exacerbating myocardial injury and cardiac systolic/diastolic dysfunction 66, whereas activation of mitophagy using Tat Beclin1 reduces mitochondrial dysfunction, improves regulation of mitochondrial energy metabolism and fatty acid oxidation, reduces lipid accumulation, prevents cardiac diastolic dysfunction and reduces metabolic myocardial injury 67. In addition, PINK1-Parkin-mediated mitophagy can shift mitochondrial substrate preference from carbohydrate to fatty acid 68.

Hexokinase II (HK-II) catalyzes the first step in glucose metabolism in cardiomyocytes and is deeply involved in mitochondrial energy metabolism. Moreover, HK-II also acts as a signaling molecule that suppresses cell death mediated via the mitochondrial pathway. During myocardial ischemia, HK-II serves as a sensor of metabolic disorder that enhances mitophagy to maintain basic energy metabolism and resist stress-induced mitochondrial damage 69. Dissociation of HK-II from mitochondria under ischemic conditions induces mitochondrial recruitment and activation of parkin and enhances ubiquitination of mitochondrial proteins, thereby increasing levels of mitophagy and protecting against cell death 70. However, the loss of mitochondrial HK-II under ischemia disrupts the stability of mitochondrial contact sites and leads to the degradation of BcL‑xL, increased permeability of the outer mitochondrial membrane, and release of cytochrome C. In addition, during reperfusion, the loss of HK-II stimulate ROS production and the opening of mPTPs, resulting in myocardial injury 71.

### MQC in cardiomyocyte hypertrophy and fibrosis

Cardiomyocyte hypertrophy refers to increases in cardiomyocyte volume, diameter, width or length and the number of sarcomeres. Cardiomyocyte proliferation occurs when myocardial hypertrophy is excessive, which a key reason for myocardial hypertrophy and heart failure seen in the late stages of ICM 72. Cardiomyocytes are terminal cells with high contraction and expansion characteristics. They generally do not independently proliferate, but able to increase in size a hypertrophic state.

The contractile proteins within cardiomyocytes include myosin, actin and tropomyosin. Experiments show that when the heart is stimulated by increased load, or the heart is hypertrophic, long-term excessive pressure and/or load can lead to contractile protein dysfunction and increased ventricular wall stress, resulting in myocardial hypertrophy 73. Increased energy consumption induced by stress or myocardial dysfunction alters cell metabolism to increase mitochondrial work, which can trigger secondary diseases and worsen ICM, eventually leading to end-stage cardiomyocyte hypertrophy. Evidence shows that patients with myocardial hypertrophy and animal models of myocardial hypertrophy show excessive accumulation of mitochondrial ROS and decreased endogenous antioxidant enzyme activity. This is accompanied by mitochondrial dysfunction involving disruption of mitochondrial oxidative phosphorylation and mitochondrial protein homeostasis and 74.

In 3-week- and 6-month-old cardiac hypertrophy model mice, mitochondria exhibit an abnormal structure with increased numbers of smaller than normal mitochondria. Expression of mitochondrial DNA coding genes is inhibited, and mitochondrial ATP is reduced 75. In addition, expression of the mitochondrial biosynthetic proteins PGC-1α and NRF1 is significantly inhibited in the mouse cardiac hypertrophy models 76. This confirms the involvement of MQC in myocardial hypertrophy. Therefore, the interaction of both PGC1a and Nrf1/2 may have important regulatory effects on mitochondrial biogenesis and mitochondrial homeostasis. And the two can further affect the development and maturation of cardiomyocytes and the synthesis of mitochondrial mtDNA, and regulate the life process of cardiomyocytes.

MQC disorders not only lead to cardiomyocyte hypertrophy, but also contribute to myocardial fibrosis 77. Studies have shown that the level of mitochondrial fragmentation is increased in cardiac fibroblasts treated with TGF-β1. In addition, expression of mitochondrial fission-related proteins Mff\Fis1 and Drp1 is significantly up-regulated, and levels of the fusion-related proteins opa1 and mfn1 is decreased. Inhibition of mitochondrial division significantly reduces TGF-β1- induced activation of cardiac fibroblasts. TGF-β1 also increases cellular levels of ROS and triggers Pink1-mediated mitophagy, and this effect is weakened by application of antioxidants. Mitochondrial fission also increases glycolysis, which plays a key role in the activation of cardiac fibroblasts 78. Similar results are seen in primary mouse aortic adventitial fibroblasts. In those cells, Drp1 dephosphorylation induces mitochondrial fission and stimulates the proliferation, migration and phenotypic transition of AngII-treated outer membrane fibroblasts. HSP90 inhibit Drp1 dephosphorylation and significantly reverses Ang-II induced mitochondrial fission and fibroblast proliferation and migration 79. The AngII-induced damage to myocardial mitochondria is related to an imbalance of mitochondrial redox. By comparing the effect of overexpression of mitochondrial targeted catalase (mCAT) with overexpression of peroxisome targeted catalase (pCAT) in mice, it can be shown that AngII-induced cardiac hypertrophy, fibrosis and mitochondrial damage is somewhat less in mCAT-overexpessing mice than WT mice 80.

### MQC and cardiomyocyte inflammation

Cardiomyocyte inflammatory response plays an important regulatory role in the pathological mechanism of ischemic cardiomyopathy. It can directly affect the apoptosis or necroptosis program of cardiomyocytes and cause severe myocardial damage. We have previously reported the important regulatory role of inflammasome NLRP3 and mitochondria in ICM. In a mouse model of heart failure with preserved ejection fraction (HFpEF), mitochondria exhibit increased acetylation, and NLPR3 inflammasome assembly is increased on the hyperacetylated mitochondria. In addition, excessive production of IL-1β/IL-18 and severe myocardial fibrosis also appeared. Increases in β‑hydroxybutyric acid levels can attenuate the NLPR3 inflammasome formation, mitochondrial dysfunction and myocardial fibrosis caused by overexpression of pro-inflammatory cytokines. The interaction of mitochondrial hyperacetylation and inflammation is a key driver of the pathogenesis of HFpEF 81.

Studies have also shown that mitochondrial DNA released from autophagic cardiomyocytes promotes inflammatory responses mediated via Toll-like receptor (TLR-9) and can induce severe myocardial damage. Consistent with that finding, the myocardium of DNase II-deficient mice showed inflammatory cell infiltration and increased expression of inflammatory cytokine messenger RNA, and autolysosomes within the myocardium show deposition of mitochondrial DNA. TLR-9 knockout reduces the severity of the myocardial inflammation seen in DNase II-deficient mice 82.

MQC also indirectly affects inflammatory responses in rat cardiomyocytes. During LPS‑induced inflammation, STE20-like protein kinase 2 (MST2) mediates the up-regulation the mitochondrial fission genes Drp1, Mff and Fis1. This effect was associated with leading to reductions in mitochondrial ATP production, dysregulation of mitochondrial respiratory chain, increased mitochondrial oxidative stress, loss of membrane potential, and increased mitochondrial fragmentation. In addition, expression levels of IL-2, IL-8, MMP9, and Caspase-3/9 mRNA and protein are all increased. Also contributing to these effects was the interaction between Drp1 and Fis1 can alter mitochondrial dynamics within cardiomyocytes. Pretreatment with the MST2 inhibitor XMU-MP1 or inhibition of the Drp1-Fis1 interaction significantly reduces the mitochondrial fission and release of inflammatory factors, enabling mitochondrial homeostasis and cardiomyocyte activity to be maintained 83.

The studies summarized above indirectly confirm the regulatory function of MQC in cardiomyocytes under inflammatory stress, though the regulatory functions of mitophagy and the UPR^mt^ under those conditions have not yet been elucidated. In a preliminary study, we found that mitophagy and the UPR^mt^ enable mitochondrial energy metabolism and respiratory chain function to be maintained under stress, ensuring an energy supply for cardiomyocytes. It has also been observed that in LPS-treated cardiomyocytes, urolithin A increases mitophagy, reduces mitochondrial fragmentation, restores the mitochondrial membrane potential, inhibits excessive production of mitochondrial ROS, and significantly reduces the inflammatory injury of cardiomyocytes 84. After FUNDC1 knockout, however, mitophagy is inhibited, and the protective effect of urolithin A is blocked.

Interestingly, enhancement of endogenous UPR^mt^ by oligomycin reduces the mitochondrial injury and myocardial dysfunction. After FUNDC1 knockdown using targeted siRNA, mitophagy was inhibited, but UPR^mt^ was not blocked. We therefore suggest that endogenous UPR^mt^ is a downstream regulator of mitotic phagocytosis, serving as a supplementary regulator to maintain mitochondrial homeostasis and suppress myocardial inflammatory injury when mitophagy is inhibited. Mitophagy and UPR^mt^ may thus play synergistic regulatory roles after inflammatory injury to cardiomyocytes and serve as complementary regulators protecting mitochondrial structure and function in cardiomyocytes under inflammatory stress 85.

Mitochondrial biogenesis may also play a regulatory role after inflammatory injury to cardiomyocytes. Sirtuin-3 (SIRT3) and AMPK indirectly stimulate mitochondrial biogenesis, thereby increasing mitochondrial renewal and cardiomyocyte functionality. In LPS-treated cardiomyocytes, the inflammatory responses are associated with decreases in SIRT3 and AMPK levels, which are followed by mitochondrial respiratory chain dysfunction, mitochondrial oxidative stress, and abnormal opening of mPTPs, as well as inhibition of PGC1α, Tfam and NRF2 expression and activation of caspase‑3. Conversely, overexpression of SIRT3 activates AMPK, improves mitochondrial redox balance and respiratory chain function, and increases expression of PGC1α, Tfam and NRF2 86. These findings suggest MQC is a key mediator of myocardial cell damage in cardiomyocytes subjected to inflammatory stress. In another study we found that PGC-1α knockout exacerbated neuropathy, whereas overexpression of TFAM had a protective effect on mitochondria. It can be seen that PGC-1α and TFAM can jointly regulate mitochondrial biosynthesis and turnover, which in turn affects mtDNA replication and mtDNA depletion 87.

### MQC in cardiomyocyte oxidative stress and calcium homeostasis

In hears affected by ICM, cardiomyocytes produce large amounts of extracellular matrix and ROS. The oxidative stress caused by the excessive accumulation of ROS leads to alterations in cell membrane permeability and, in turn, Ca^2+^ overload, expression of inflammatory factors, and cell apoptosis. Because the myocardium is rich in mitochondria and mitochondria are the main producers of ROS 88. ROS produced by mitochondria are important contributors to cardiomyocyte injury, and are a principle factor affecting mitophagy and mitochondrial fusion/fission 89.

**Figure 4:**
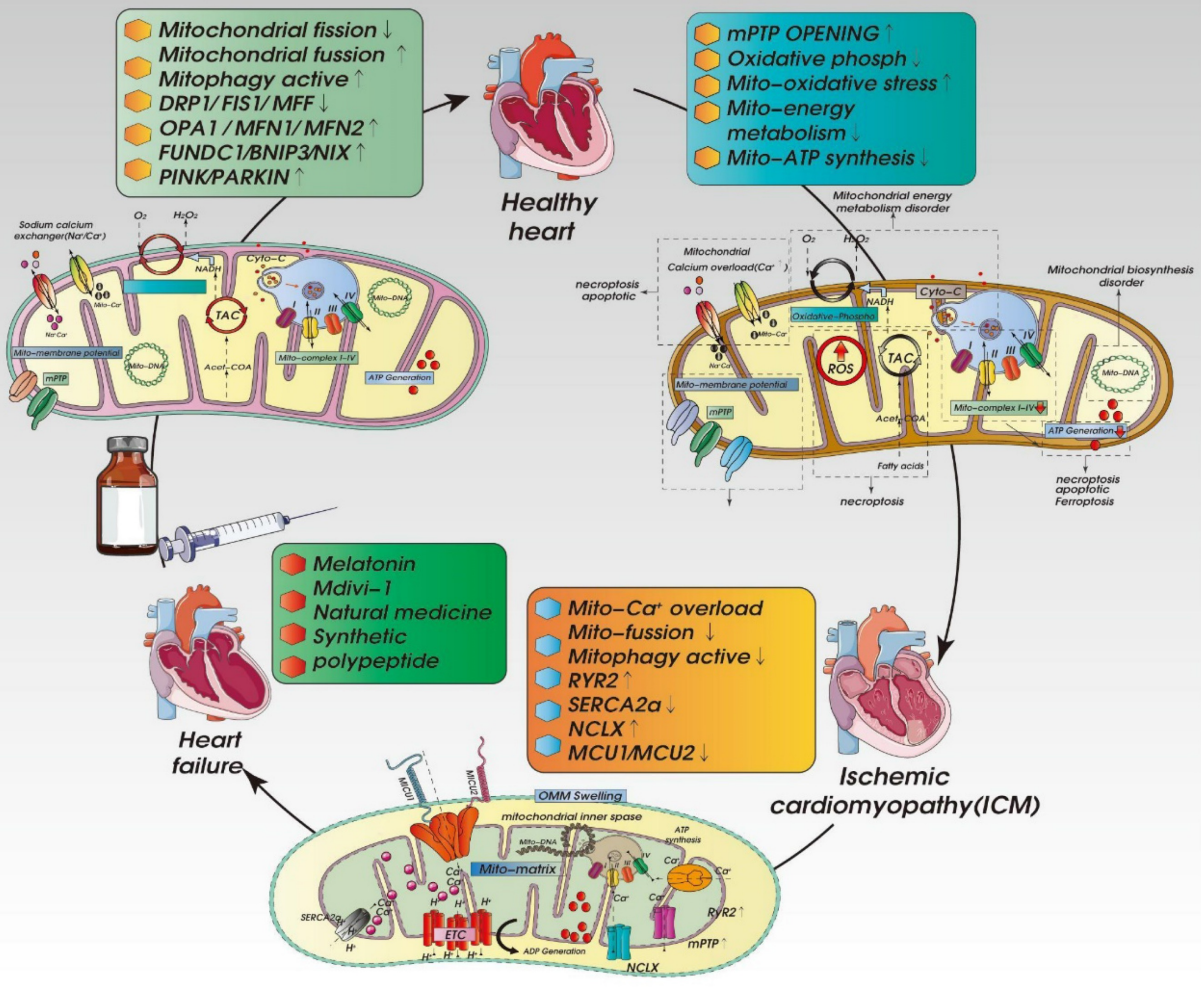
** Mechanisms of mitochondrial calcium overload-mediated oxidative stress injury and dysregulated mitochondrial.** The mitochondrial calcium homeostasis mechanism is controlled by the mitochondrial calcium uptake and release system. Sarcoplasmic/Endoplasmic Reticulum Ca 2+ -ATPase (SERCA) is a pump that transports calcium ions from the cytoplasm to the ER. The activation of SERCA can inhibit the excessive release of calcium, thereby inhibiting the excessive production of mitochondrial ROS and improving mitochondrial function. At the same time, mitochondrial homeostasis disorder caused by mitochondrial calcium overload will further induce mitochondrial PTP activation and mitochondrial energy metabolism dysfunction, leading to excessive mitochondrial division and mitochondrial quality control network disorder.

As shown in Figure 4, during prolonged mitochondrial dysfunction, the damaged mitochondria are eliminated through mitophagy, and an imbalance in the mitophagic pathway can lead to a redox dysregulation with excessive ROS production and reductions in cell energy metabolism and ATP production. Members of the mitochondrial respiratory chain, including the NOX family and NADPH oxidase, are the main sources of ROS in cardiomyocytes. Interactions among several MQC mechanisms affect the mitochondrial oxidative stress response and become the main regulatory mechanism leading to cardiomyocytes death 37, 89.

In patients with late stage acute myocardial ischemia as well as mice with heart failure induced by TAC, AMPK2α is converted to AMPK1α. In addition, PINK-mediated reductions in mitophagy and mitochondrial function are accompanied by oxidative stress damage and cardiomyocyte apoptosis. By increasing PINK-mediated mitophagy and improving mitochondrial function, cardiac overexpression of AMPK2α prevents the development of TAC-induced heart failure. By contrast, AMPK2α-/- mutant mice showed early progression of TAC-induced heart failure that was worsened by reducing cardiac mitophagy. Increasing cardiac mitophagy resulted in elimination of damaged mitochondria, improved mitochondrial function, and reductions in ROS production and cardiomyocyte apoptosis. It appears that AMPK2α- and PINK1-mediated mitophagy is a crucial regulatory mechanism that inhibits the progression of heart failure 90.

After 8 minutes of KCl-induced cardiac arrest in C57BL/6 wild-type mice, Drp1 translocates to the mitochondrial membrane, resulting in increased mitochondrial fission, and lactate and ROS production, and oxidative damage. Administration of Mdivi-1 during cardiopulmonary resuscitation can inhibit Drp1 activation, preserve mitochondrial morphology, reduce oxidative damage, and enhance myocardial function after spontaneous circulation is restored 91. It thus appears that Drp1- and Mfn-mediated mitochondrial division and fusion directly and indirectly affect cardiomyocyte oxidative damage, and their dual regulation plays a leading role in mitochondrial homeostasis.

It also appears that insulin-like growth factor II (IGF-II), acting via its receptor IGF-IIR on cardiomyocytes, can also trigger mitochondrial dysfunction, mitochondrial ROS accumulation, oxidative stress damage, and mitochondrial disruption that leads to the formation of autophagosomes. The loss of mitochondrial contents increases levels of Parkin-dependent mitophagy and initiates the cell death program via the cytochrome c pathway 92. This suggests that while excessive mitophagy can cause oxidative stress damage to cardiomyocytes, moderately upregulated mitophagy can protect cardiomyocytes from oxidative stress-induced damage. In HL-1 mouse atrial cardiomyocytes and AC16 human ventricular cells subjected to antimycin A-induced mitochondrial oxidative stress damage there was accumulation of mitochondrial ROS, and mitochondrial respiratory dysfunction. In addition, the nuclear DNA experienced oxidative damage, which triggered the cell death program. However, the mTOR inhibitor rapamycin, which suppresses antimycin A-induced protein ubiquitination induced mitophagy and promoted removal of some structurally damaged mitochondria, protecting the cells from oxidative stress 93.

MQC is also involved in the regulation of Ca^2+^ homeostasis. It is known that increases in intracellular Ca^2+^ can lead to activation of phospholipases, degradation of membrane phospholipids and increases membrane permeability that enable extracellular Ca^2+^ to flow into the cell down its concentration gradient and exacerbate Ca^2+^ overload 63. Moreover, the mitochondrial dysfunction would decrease ATP production, suppressing the ATP-dependent activity of the plasma membrane Ca^2+^ pump and SERCA, thereby inhibiting removal of the excessively high cytosolic Ca^2+^ 61, 63.

The distribution of Mfn2 within cells is not limited to the outer mitochondrial membrane, as it has also been detected in the ER membrane. The dual positioning of Mfn1 in the ER and mitochondrial membranes is thought to facilitate Ca^2+^ transfer from the ER to the mitochondria, which could potentially cause mitochondrial Ca^2+^ overload 94. Mfn2 knockdown using targeted siRNA disrupts ER morphology and the interaction between the ER and mitochondria, reducing the efficiency of mitochondrial Ca^2+^ uptake. This suggests that Mfn2 is a key mediator of Ca^2+^ transport between the ER and mitochondria and that Mfn2 is also an important regulator involved in maintaining Ca^2+^ homeostasis in cardiomyocytes under stress, which may explain why under some conditions Mfn2 mediates Ca^2+^ overload and cell death. Mfn2-deficient mice exhibit moderate cardiac hypertrophy with slight deterioration in cardiac function. However, the lack of Mfn2 increases the likelihood of Ca^2+^ overload, abnormal opening of mPTPs, changes in mitochondrial structure or morphology, and cell death 95.

### MQC and cardiomyocyte longevity regulation

Cell death is the process by which multicellular organisms clear infected, damaged, or dysfunctional cells, and a way for the body to renew itself. This process can help a healthy myocardium clear damaged cells. However, the stressed myocardium, MQC disorders (such as excessive mitophagy) also can activate the cardiomyocyte death program 96, 97. The mitochondrial cell death pathway starts from permeabilization of the mitochondrial outer membrane. Proteins such as cytochrome c and other molecules are released from the gap between the inner and outer mitochondrial membranes into the cytosol, which activates caspase-3/-9/-12 and leads to rapid cell death. Through MQC, mitophagy, fusion/fission, and mitochondrial biogenesis are all involved in regulating the fate of cardiomyocytes 98.

MQC is able to fully participate in the process of cardiomyocyte death (including apoptosis or necrosis). Under stressful conditions such as ischemia/hypoxia intracellular ROS will be overproduced and Ca^2+^ overload may occur. The balance between mitochondrial fusion and fission is disrupted, and Drp1 drives mitochondrial fission. In cardiomyocyte-specific Drp1 KO mice, mitochondria are elongated and there is accumulation of damaged mitochondria due to a decrease in mitophagy, which leads to increases in apoptosis. The severe mitochondrial dysfunction is also accompanied by left ventricular dysfunction, with some mice dying within 13 weeks 99.

It was also found that the specific deletion of Mfn2 from cardiomyocytes leads to accumulation of autophagic lysosomes and fusion of autophagosomes and lysosomes, while cardiac-specific Mfn2 KO mice exhibit dysregulation of mitochondrial energy metabolism. In addition, Mfn2 knockdown using targeted siRNA prevented autophagic lysosomal fusion in cardiomyocytes and increased the cells' susceptibility to ischemia-reperfusion. This suggests that not only does Mfn2 regulate mitochondrial fusion/fission, it also supports mitophagy in cells subjected to ischemic stress by mediating mitophagic lysosome fusion 100.

In addition to disrupting mitochondrial structure and function, hypoxia/ischemia induces AMPK activation. Stimulation of AMPK activity using the AMP analog AICAR increases mitophagy and blocks the mitochondrial apoptosis pathway. Conversely, blocking AMPK activation suppresses mitophagy and increase of cardiomyocyte apoptosis 101. Under conditions of hypoxic/ischemic stress mitochondria will reorganize its structure and function to repair/remove damaged mtDNA and dysfunctional mitochondria, as mitochondrial fusion and fission directly affects the distributions of mitochondrial proteins and DNA. When mitochondria undergo fission into two daughter mitochondria. One has a high membrane potential and high fusion affinity, while the other has a lower membrane potential and low fusion affinity 102. Investigation of mitochondrial fusion in the heart tube of Drosophila melanogaster revealed that heart-specific deletion of MARF [tinc Δ 4gal4 directional expression of mitochondrial assembly regulator] and OPA1 increases the heterogeneity of mitochondrial morphology and promotes systolic dysfunction. Moreover, inhibition of mitochondrial fusion increases compensatory expression of mitochondrial nuclear coding genes and the level of mitochondrial biogenesis 103.

We previously showed that FUNDC1 is a mitophagy receptor that interacts with LC3II to mediate mitophagy and regulate the number and quality of mitochondria in response to hypoxia‑induced cardiomyocyte injury. Under hypoxic stress, FUNDC1 can triggers mitophagy in platelets from wild-type mice. In cardiac-specific FUNDC1 KO mice, cardiac function is reduced and infarcted areas are larger following myocardial infarction. Hypoxic preconditioning can induce FUNDC1-dependent mitophagy and suppress cardiomyocyte death 104. We also found that by regulating MQC, the serine/threonine kinase casein kinase 2α (CK2α) and DUSP1 can alter mitochondrial structure and function and promote or block cardiomyocyte apoptosis or necrosis via the mitochondrial pathway. CK2α induces FUNDC1 inactivation through a post-transcriptional modification at Ser13, thereby inhibiting mitophagy, disrupting mitochondrial homeostasis, promoting cardiomyocyte death and expansion of myocardial infarction area, and cardiac insufficiency. It can also lead to dysregulation of the mitochondrial gene transcriptome, inhibition of the electron transport chain, and suppression of mitochondrial biogenesis. With the resultant accumulation of ROS and abnormal opening of mPTPs, Drp1- and Fis1- mediated mitochondrial fission is up-regulated and Mfn- and OPA1-mediated mitochondrial fusion is inhibited, increasing the numbers of fragmented mitochondria. In addition, mitochondrial DNA is destroyed, which ultimately leads to mitochondrial apoptosis 105. However, deletion of CK2α increases FUNDC1-mediated mitophagy, restores balance to mitochondrial fusion/fission, increases the level of mitochondrial biogenesis and the copy number/transcription of mtDNA, and inhibits cardiomyocyte apoptosis 105.

DUSP1 expression is down-regulated after acute cardiac ischemia/reperfusion injury. Compared to wild-type mice, DUSP1 transgenic mice also show a smaller infarct size and better cardiac function. DUSP1 deficiency promotes JNK activation, which enhances expression of Mff and promotes phosphorylation/activation of Bnip3. This increases mitophagy levels, and a large number of mitochondria to be cleared, resulting in insufficient mitochondrial ATP production and activation of caspase-3/-9-mediated cardiomyocyte apoptosis. Interestingly, reintroduction of DUSP1 attenuates Mff/Bnip3 activation, inhibit the mitochondrial fission and mitophagy, and reduce cardiomyocyte apoptosis 106.

### MQC in vascular degeneration, stiffness and fibrosis

During the onset of ICM, the structure and function of coronary blood vessels undergo pathological changes that include thickening and hardening of arterial walls, dilatation of the vascular lumen, thickening of the arterial intima, and vascular calcification 107. Under ischemic/hypoxic stress, the vascular collagen content increases while the elastin content decreases, which decreases the stretchability of the arterial wall. Eventually, the arterial blood supply function is reduced, and the myocardium becomes ischemic/hypoxic, which causes a decline of heart function and the occurrence of heart failure. Vascular endothelial dysfunction and arteriosclerosis are independently associated with an increased risk of ICM. These are therefore major therapeutic targets for ICM prevention and treatment 108, 109.

Under physiological conditions, vascular cells, and especially vascular endothelial cells, are a dynamic balance of regeneration, growth, degradation and death. In the process of cardiovascular disease or aging, blood vessels are degenerating and dying, and the ultrastructure of vascular endothelial cells are significantly altered. Aging vascular endothelial cells become flattened and widened, cell gaps also widen, intracellular ROS production increases, cell permeability increases, and macromolecular substances enter the intima from the plasma, resulting in abnormal endothelial cell function and impaired endothelium-dependent vasodilation, which eventually leads to vasospasm, thrombosis, macrophage infiltration, and inflammatory responses 110.

**Figure 5:**
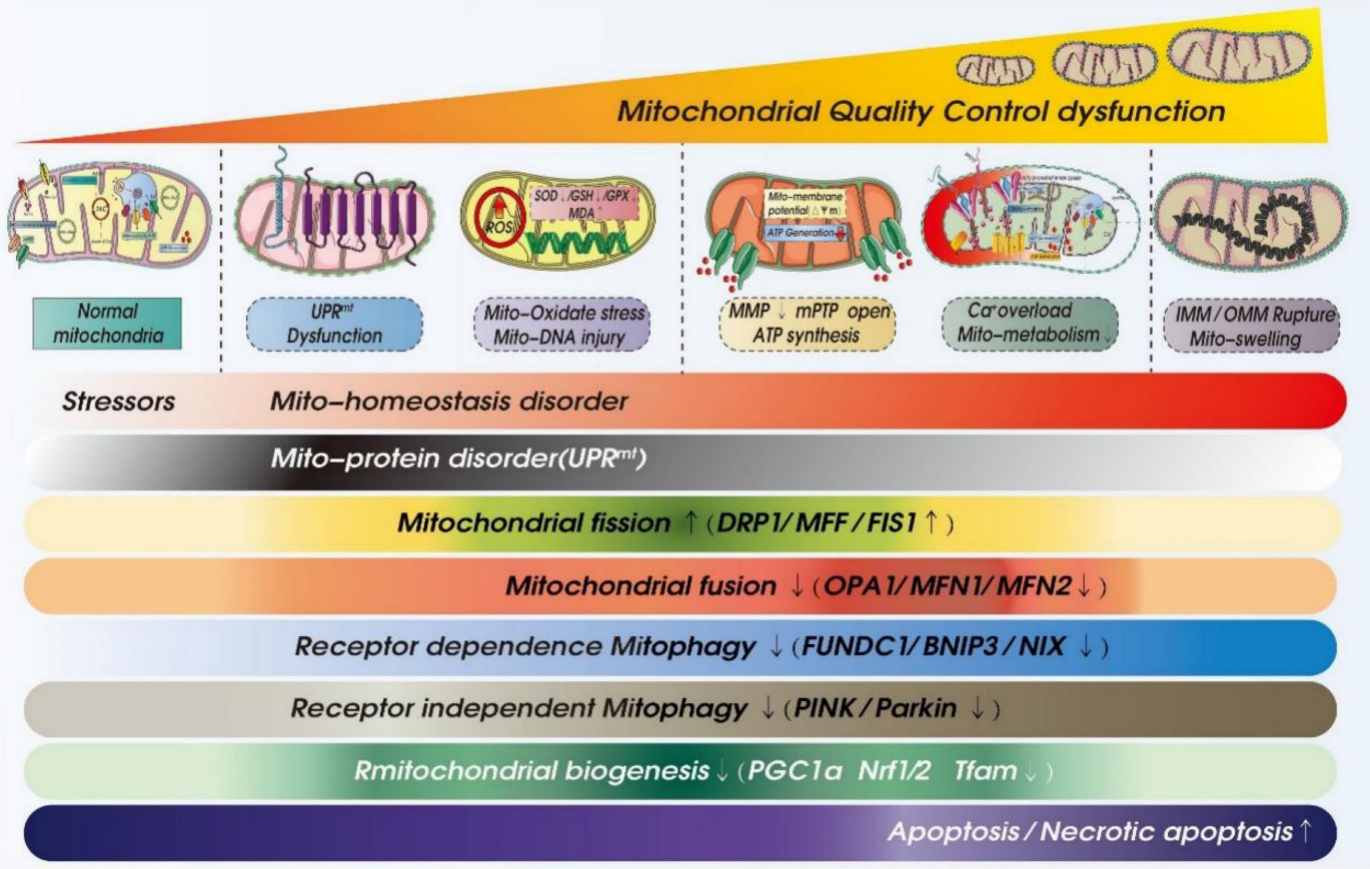
** Mitochondrial and myocardial/coronary microvascular endothelial cell injury.** Mitochondrial injury is accompanied by reduced ATP production and oxidative stress during coronary microvascular injury in end-stage IM and ICM. This stimulates increased levels of mitochondrial fragmentation. Excessive stress stimulation affects mitochondrial function and also leads to mitochondrial DNA damage, which can damage the transcription and translation of mitochondrial electron transport chain proteins and induce mitochondrial fusion dysfunction. Excessive mitochondrial damage and stress will promote mitochondrial division, thus transforming the reticular mitochondrial network into point and fragment mitochondria. However, FUNDC1 and PINK/Parkin mediated mitophagy cannot completely remove fragmented mitochondria, which will lead to dysfunction of mitochondrial energy metabolism, which will reduce ATP production and affect the life of myocardial cells. Furthermore, PGC1α- and Tfam-mediated mitochondrial biosynthesis dysfunction also affects mitochondrial neogenesis. lead to the death of coronary microvascular endothelial cells.

Mitochondria are present in all vascular cell types, where they play important roles regulating tissue repair, apoptosis, and a variety of physiological and pathological processes. As shown in Figure 5, in cardiomyocytes, excessive accumulation of mitochondrial ROS, impaired MQC and mitochondrial Ca^2+^ overload can lead to vascular injury 111. However, different from mitochondria in cardiomyocytes, where there is high energy demand, the content of mitochondria in vascular endothelial cells accounts for only 2%-6% of the cytoplasmic volume, and their main function is to transmit the cells' responses to environmental signals 112. In the pathogenesis of ICM, vascular endothelial dysfunction is closely related to mitochondrial structural and functional dysfunction. MQC is involved in the occurrence and development of vascular aging, arteriosclerosis and arterial fibrosis 112. These processes interact with metabolism and energy demand in endothelial cells.

Activation of mitochondrial fission mediated by Drp1 and excessive production ROS in endothelial cells cause oxidative stress and endothelial dysfunction 113, 114. With activation of Drp1 and excessive mitochondrial production of ROS, the level of mitochondrial fragmentation is increased. However, Drp1 knockdown using targeted siRNA or treatment with a mitochondrial fission inhibitor improves endothelial cell function, enables mitochondrial structure and redox balance to be maintained, and improves endothelial cell activity 115. This makes regulation of mitochondrial fusion/fission key to the maintenance of endothelial cell function. Similar findings were also made in the vascular endothelium of spontaneously hypertensive rats, the thoracic aortas of which exhibit increased medial thickness and elevated expression of IL-6/TNF-α and Drp1. After administration of Mdivi‑1, however, inhibition of excessive mitochondrial fission reduces inflammatory responses and protects the vascular endothelium 116.

Selective mitophagy can reverse endothelial-dependent relaxation dysfunction that increases arterial stiffness in aging blood vessels by altering the total redox state of the vessels through inhibition the ROS production. Mitophagy inhibition exacerbates the vascular injury caused by oxidative stress, and interaction of fibrosis with mitochondrial fission inhibition is a key factor leading to vascular remodeling 117. Protective mitophagy can delay the progression of vascular calcification, while lactate can induce expression of NR4A1 and activation of DNA-PKCs/p53. Inducing Drp1 to migrate to mitochondria in human aortic smooth muscle cells increases mitochondrial fission and fragmentation, inhibits BNIP3-mediated mitophagy, disrupts mitochondrial energy metabolism, and leads to abnormal opening of mPTPs and loss of mitochondrial membrane potential. These effects promote apoptosis among aortic smooth muscle cells and accelerates the Ca^2+^ deposition and vascular calcification. We also observed that in WT mouse endothelial cells, NR4A1 activates CK2α, promotes Mff phosphorylation, and enhances translocation of Drp1 to mitochondria, leading to excessive mitochondrial cleavage and disruption of mitochondrial structure and function and, ultimately, accumulation of damaged mitochondria and apoptosis. Deleting NR4A1 reverses these effects, enabling stable mitochondrial structure and function to be maintained and protecting against acute microvascular injury, which enabled vascular function and endothelial cell activity to be maintained 118. These findings further confirm the important protective regulatory roles of MSQ, mitochondrial fusion/fission, mitophagy and mitochondrial biogenesis in vascular endothelial cells.

### MQC in cardiac microvascular endothelial cell regeneration, proliferation, mobilization and paracrine function

The cardiac microvasculature is composed of coronary arteries with diameters ranging from 100-400 μm and capillaries with diameters less than 10 μm 119. Unlike the main coronary blood vessels in the heart, coronary microvessels cannot be examined with coronary angiography. However, because microvessels account for 95% of the coronary circulation, and microvascular damage or disorder cannot be detected by early coronary angiography, coronary microvascular disease is clinically regarded as an “underwater iceberg” and a non-negligible contributor to the pathogenesis of ICM 120. The primary function of the cardiac microvascular system is to distribute blood flow and play an important auxiliary role in regulating blood circulation. As the end of coronary efferent circulation, coronary microvessels can directly affect myocardial perfusion. Indeed, the impact of coronary microvascular dysfunction on myocardial ischemia is now receiving clinical attention 119, as coronary microvascular disorders cannot be resolved through percutaneous coronary intervention (PCI) or coronary artery bypass graft, and there are no drugs that specifically target coronary microvascular ischemia or dysfunction. When patients experience persistent chest pains or other ischemic symptoms without obvious coronary atherosclerosis or continue to show slow blood flow after PCI, these symptoms are currently believed to be related to coronary microvascular disease 121.

In ICM patients, microvascular damage generally occurs in necrotic or infarcted areas within the myocardium, so re-establishing blood flow may not yield improvement of the patient's condition. However, restoring flow can enable transmission of nutrients to the necrotic area, speeding up healing, reducing infarct expansion, delaying left ventricular remodeling, and promoting formation of new microvessels 122. Moreover, mechanical stimulation of the vascular wall and vasoactive substances in the blood, and release to make stress response to external stimulation and unknown source information.

Microvascular endothelial cells cover the inner wall of blood vessel and are in direct contact with the bloodstream. They are usually in a differentiated and static state but under the action of angiogenic factors, they change from a differentiated to an undifferentiated state. The functions of microvascular endothelial cells include reducing vascular permeability, regulating material exchange between tissue and blood, maintaining blood fluidity by balancing anticoagulant/fibrinolytic systems and antiplatelet functions, and regulating vasoconstriction/relaxation by releasing vasoconstrictor and diastolic factors 123. The energy demand in endothelial cells is relatively low, and glycolysis is the main source of ATP production. In cultured endothelial cells, at least 75% of ATP synthesis depends on glycolysis, and only a small part depends on mitochondria. Nevertheless, PGC-1α is abundantly expressed in endothelial cells, where it participates in mitochondrial biogenesis, exerts anti-apoptotic and anti-inflammatory effects, and improves the bioavailability of NO. Overexpression of PGC-1α increases expression of uncoupling protein 2 (UCP2), MnSOD, CAT and other antioxidant enzymes 124, 125. In that way, PGC-1α contributes to the maintenance of mitochondria and repair of oxidative damage in microvascular endothelial cells 126. Also involved are mitochondrial fusion\fission, mitophagy and other regulatory mechanisms.

In an earlier literature summary, we explained that RIPK3-mediated apoptosis is the primary factor contributed to the pathogenesis of myocardial ischemia-reperfusion injury. RIPK3 is also an important regulator endothelial cell apoptosis. Following ischemia-reperfusion, vascular endothelial expression of RIPK3, IP_3_R, and XO is enhanced, which leads to oxidative stress injury, Ca^2+^ overload, and mitochondrial swelling/deformation, and eventually vascular endothelial cell apoptosis initiated via the mitochondrial pathway 127.

In an experimental study of myocardial ischemic injury, we confirmed that RIPK3 migrates to SR, causing in stress injury in cardiomyocytes. It is therefore likely that the endothelial Ca^2+^ overload is related to RIPK3-mediated ER stress. Deletion of RIPK3 reduces expression of adhesion molecules and various inflammatory factors and reduces endothelial swelling and apoptosis, which enables the patency of the microvascular system to be maintained 127. In addition, we showed that overexpression of SERCA can normalize the ratio between eNOS and ET-1 levels in cardiac microvascular endothelial cells, restore intracellular Ca^2+^ homeostasis, and inhibit abnormal activation of the MCU and opening of mPTPs 128. This significantly reduced vascular wall edema and lumenal stenosis. Moreover, SERCA overexpression enhanced Mfn2- and OPA1‑mediated mitochondrial fusion, parkin-dependent mitophagy, PGC1‑α‑mediated mitochondrial biogenesis, and the level of mitochondrial DNA synthesis, which corrected the MQC imbalance and protected microvascular endothelial cells following ischemia/reperfusion 129.

## Regulation of MQC for Management of ICM

### Lifestyle modification, exercise, and smoking cessation

For ICM patients, poor lifestyle is an important factor that affects the pathological mechanism and pathogenesis of the disease. As mentioned before, sedentary lifestyles, smoking and other bad lifestyles can to varying degrees directly and indirectly cause vascular damage or blockage, becoming important pathogenic factors for ICM 130. The recent decline in ischemic heart disease mortality is directly related to lifestyle changes. Research has found that about 40% of the decline in ICM deaths can be attributed to medical interventions and lifestyle interventions. Because many of these interventions have not yet reached their full potential in terms of the universality of the intervention protocol and acceptance by the public, it is expected that the mortality rate from ICM will continue to decline over the next few decades 131. However, for this to occur it will be necessary for ICM patients to change their exercise habits, though the persistence of myocardial ischemia is determinant to optimization of exercise plans. Exercise training is an important auxiliary treatment for cardiac rehabilitation of ICM patients, which can reduce ICM mortality, morbidity and re-hospitalization rates, and can alleviate the development of myocardial hypertrophy and heart failure at later ICM stages 132.

From the perspective of mitochondrial redox balance, exercise or training can increase the activity of antioxidant enzymes and improve the ability of GSH, GST and GSH-Px to scavenge free radicals 133. However, increasing exercise intensity and extending exercise duration can potentially cause a rapid increase of ROS production and resulting lipid peroxidation, which would be damage to the body, so care must be taken 134. Studies have also shown that exercise inhibits and delays the progression of mitochondrial dysfunction by improving mitochondrial metabolism, biosynthesis, and respiratory chain function. Moreover, exercise affects post-translational modification of MQC-related proteins, enhances the function of mitochondrial kinetic regulatory proteins, and indirectly modifies the regulation of the mitochondrial fission/fusion balance and mitophagy 135.

Exercise has beneficial effects on age-related damage to mitochondrial biogenesis and dynamics and reductions of mitophagy. As a result, exercise may have an impact on crucial cell signaling pathways involved in maintaining mitochondria quality and quantity in the elderly. Although several exercise programs are known to improve mitochondrial structure and function, further research is needed to better understand the cell signaling pathways involved in the control of mitochondrial quality and quantity. Several exercise programs are known to modify mitochondrial activity and turnover, but additional study is needed to precisely determine the interaction mechanism between mitochondrial function, aging and physical activity, as well as the factors affecting mitochondrial function 136.

From the perspective of MQC, exercise maintains mitochondrial function and cardiomyocyte activity by promoting the clearance of dysfunctional mitochondria through mitochondrial biogenesis and mitophagy. Moderate exercise can cause mitochondrial oxidative stress and promote the mitophagy to remove damaged or dysfunctional mitochondria and maintain cell homeostasis. Moderate and heavy exercise have a two-way regulatory effect on mitochondrial fusion and fission 137. During strenuous exercise in rats, Mfn1 and Mfn2 mRNA transcription and protein expression gradually decreases, while Fis1 expression and mitochondrial fragmentation increase. This may be due to the strong cellular demand for ATP during vigorous or prolonged exercise. Under those conditions, mitochondria may divide to produce more mitochondria to meet demand, but mitochondria with a split structure not only cannot produce enough ATP, but also affect the energy metabolism of the normal mitochondria 135, 138. Exercise is thus a complex stimulus, and it may be possible to achieve two-way regulation of cardiac function by regulating the MQC governing myocardial mitochondria. Appropriate low- and medium-intensity exercise improve myocardial mitochondrial respiratory function as well as myocardial cell energy metabolism by up-regulating the MQC system. High-intensity exhaustive exercise may cause autophagy and even apoptosis through the MQC, reducing the energy metabolism level of cardiomyocytes 139.

### Pharmacological intervention

In recent years, research has provided variety of drugs, such as the mitochondrially targeted antioxidant MitoQ, that exert protective effects on cardiomyocytes and endothelial cells by affecting the regulation MQC and mitochondrial homeostasis and mitigating myocardial injury after infarction 140. Studies have shown that MitoQ has beneficial regulatory effects on vascular endothelial cells in elderly mice. Administration of MitoQ to elderly mice with primary aging-related endothelial dysfunction improves endothelial function and increases mitochondrial levels of PGC1a\mtSOD and COX-IV, while also reducing susceptibility to mitochondrial damage 141. Another compound, the hormone melatonin, has been shown to significantly reduce infarct area and improve heart function and blood flow restoration after myocardial infarction. Melatonin inhibits Drp1‑dependent mitochondrial fission through AMP-activated protein kinase α (AMPKα); inhibits oligomerization of voltage‑dependent anion channel 1 (VDAC1), release of hexokinase 2, and abnormal opening of mPTP; improves the activity of mouse cardiac microcirculation endothelial cells; and reduces infiltration by inflammatory cells and endothelial damage. In addition, in both acute myocardial infarction patients and ischemia/reperfusion injury model mice, melatonin is able to mitigate ischemia/reperfusion injury. However, deletion of AMPKα eliminates the beneficial effects of melatonin on the quality of cardiomyocytes and endothelial cells 142.

Melatonin is also able to influence MQC and SR homeostasis through BAP31 and reduce LPS-mediated myocardial inflammatory damage. It does this by supporting mitochondrial function and inhibiting ER/SR stress, which improves cardiomyocyte viability. On the other hand, inhibition of the ERK pathway and BAP31 reduces melatonin's ability to affect mitochondrial function and ER/SR homeostasis under LPS stress 143. Melatonin also suppresses myocardial damage caused by cardiorenal syndrome type 3 (CRS-3) following acute kidney injury. In the kidney, ischemia/reperfusion injury has the same adverse effects in the kidney as it does in the heart. That is, it inhibits the quality control of mitochondrial function, resulting in loss of mitochondrial membrane potential, mitochondrial fission/fusion balance and Ca^2+^ homeostasis, which disrupts energy metabolism. Interestingly, MCU activation eliminates the cardioprotection provided by melatonin, which results in disruption of mitochondrial structure and function in cardiomyocytes. It thus appears the negative effects of renal ischemia/reperfusion injury on myocardial vitality and cardiac function are related to IP_3_R phosphorylation, MCU upregulation, and Ca^2+^ overload. Melatonin protects heart function from CRS-3 by inhibiting IP_3_R-MCU signaling 144.

Several drugs used to treat or prevent the myocardial microvascular injury target the MQC. These include empagliflozin, a sodium-glucose cotransporter 2 (SGLT2) inhibitor that has been shown to have a cardioprotective effect. We observed that empagliflozin improves the structure and function of the diabetic myocardium, in part by inhibiting cardiac microvascular endothelial cell senescence as well as the production of mitochondrial ROS and the resultant oxidative stress, thereby protecting the barrier function of cardiac microvascular endothelial cells. Empagliflozin also supports eNOS phosphorylation and endothelial-dependent relaxation and improves microvessel density and perfusion. What's more, it triggers AMPK activation, inhibits Drp1 S^616^ phosphorylation and increases Drp1 S^637^ phosphorylation, which ultimately inhibits mitochondrial fission and restores mitochondrial function 145.

In addition, the mitochondrial fission inhibitor Mdivi-1 protects the myocardium by affecting the regulation of the mitochondrial fission/fusion. By inhibiting excessive mitochondrial fission during ischemia/reperfusion injury, Mdivi-1 preserves the integrity of mitochondrial structure and function in cardiomyocytes and reduces infarct area after myocardial infarction 146. Mdivi-1 also reduces Drp1 expression, increases Mfn2 expression, suppresses excessive inhibition of autophagy, and preserves mitochondrial respiratory chain function and oxidative phosphorylation, thereby protecting cardiomyocytes 147. Moreover, Mdivi-1 directly interacts with ion channels to maintain the stability of the intracellular electrophysiological environment in cardiomyocytes. in HL-1 cells, for example, Mdivi-1 treatment leads to increased production of spontaneous action potentials 148.

We also observed that quercetin, an active ingredient in various natural medicines, protects human cardiomyocytes subjected to hypoxia/reoxygenation. Quercetin pretreatment inhibits H/R‑mediated ROS production and oxidative stress. In addition, quercetin increases mitophagy, up-regulates transmembrane BAX inhibitor-1 motif containing 6 (TMBIM6) mRNA and protein expression, which preserves mitochondrial energy metabolism and suppresses the SR stress in human cardiomyocytes. These protective effects of quercetin are blocked by SIRT1 knockdown using targeted siRNA 149.

## Future Perspectives and Translational issues for mito-protective agents in ICM treatment

In this review, we summarized the clinical symptoms, disease manifestations at different stages, and epidemiological characteristics of ICM. The incidence of ICM is related to unhealthy lifestyle habits, including smoking, lack of exercise, and a high-fat/high-sugar diet. At present, substantial progress has been made in our understanding of ICM and mitochondrial. Although the medical community has some understanding of the mechanism underlying the abnormal mitochondrial contributing to the occurrence and development of acute myocardial injury after myocardial infarction or ischemia/reperfusion, the pathological mechanism of coronary microvascular/microcirculation injury or dysfunction after acute ischemia/reperfusion has not yet been clarified, and there is a need for further research into relevant targeted therapeutic drugs. It will involve both animal and cell experiments and translational studies that explain the mechanism underlying the clinical features of coronary microvascular injury. This highly important, as coronary microvascular injury is a crucial driver of the occurrence and development of ICM. In addition, the roles of mitochondrial biosynthesis and the mitochondrial unfolded protein response in ICM is not well understood. It will therefore be necessary to conduct in-depth exploration of the mitochondrial quality detection system and directly connect ICM to ICM-related vascular injury. This could potentially serve as the basis for developing mitochondrially targeted strategies to improve myocardial and vascular function and provide a theoretical basis for the prevention and treatment of ICM and other vascular injury diseases.

## Summary and Conclusion

In this review, we endeavored to summarize mitochondrial quality monitoring and the pathological mechanisms responsible for the development and progression of ICM. The important contribution of abnormal MQC to the occurrence and development of vascular aging and aging-related cardiovascular diseases is now well established. At present, however, research mainly involves animal and cell experiments, though a few translational studies have shown the clinical relevance of these mechanisms to humans. In addition, little is known about the role of changes in mitochondrial dynamics in vascular aging. It is therefore necessary carry out a more in-depth exploration to establish the precise linkage between changes in MQC and vascular aging and aging-related cardiovascular diseases. It is anticipated this will provide a theoretical basis for the development of mitochondrially targeted strategies to improve vascular function as well as to prevent and treat ICM.

## Funding

This study is supported by the National Natural Science Foundation of China (No. 81902011)

## Figures and Tables

**Table 1:** Molecular Pathophysiology of ICM

Mitochondrial quality control	Regulators	Model
***Inflammation***	IL-6/ICAM-1/MMP-9/TNF- α/IL-13/TLR/NLRP3	Cardiomyocytes/endothelial cells
***Oxidative stress***	ROS/SOD/CAT/GSH/ GPX/TreX	Cardiomyocytes/endothelial cells
***Cell death***	Cyt-C/Smac/HtrA2/Omi/Caspase-3/Caspase-9/RIPK3/MLKL/CypD/CaMKII/	Cardiomyocytes/endothelial cells
***Fibrosis***	α-SMA/cyto-C/TNF- α/ERK1-2/Smad/TGF-β	Cardiomyocytes
***Calcium signal disorder***	Cyto-C/SMAC/Omi/HTR2A /AIF/MCU1-MCU2/SERCA/RyR/CaMKII	Cardiomyocytes/endothelial cells

**Table 2:** Mechanism of MQC in ICM

Mitochondrial Quality Control	Protein substrate	Full name	Regulators	Target cell
Mitochondrial fission	Drp1/DLP1	Dynamin-related protein 1	ERK (extracellular regulated protein kinases)	Cardiomyocytes/Cardiac microcirculation endothelial cells (CMECs)
	Fis1	Fission 1	TBC1D15 (TBC domain family member 15)	Cardiomyocytes
	Mff	Mitochondrial fission factor	1.Voltage-dependent anion channel 1/hexokinase 2 (HK2/VDAC1) 2. DUSP1 (Dual-specificity protein phosphatase1)	Cardiac microcirculation endothelial cells (CMECs)
Mitochondrial fusion	OPA1	Optic Atrophy 1	1. TNFα receptor 2 (TNFR2) 2. AMPKα2 (AMP-activated protein kinase)	Cardiomyocytes
	Mfn1	Mitofusin1	DNA-dependent protein kinase catalytic subunits (DNA-Pkcs)	Cardiomyocytes
	Mfn2	Mitofusin2	DNA-dependent protein kinase catalytic subunits (DNA-Pkcs)	Cardiomyocytes
Mitophagy (Receptor-dependent)	FUNDC1	FUN14 domain-containing 1	1. Serine/threonine kinase casein kinase2α (CK2α) 2. Nuclear receptor subfamily 4 group A member 1 (NR4A1)	Cardiomyocytes/Cardiac microcirculation endothelial cells (CMECs)
	BNIP3	BCL2/adenovirus E1B 19-kDa interacting protein 3	1. JNK(c-Jun N-terminal kinase) 2. Dual-specificity protein phosphatase1 (DUSP1)	Cardiomyocytes
	NIX	Nip3-like protein X		
Mitophagy (Receptor-independent)	PINK1/Parkin	PTEN induced putative kinase 1	AMP-activated protein kinase-α2 (AMPKα2)	Cardiomyocytes/Cardiac microcirculation endothelial cells (CMECs)
Mitochondrial biogenesis (Post-transcriptional regulation)	PGC-1 α/PPARγ	Peroxisome proliferator activated receptor γ /Peroxisomeproliferator-activated receptor-γ coactivator-1α		Cardiomyocytes
	NRF1/2	Nuclear respiratory factor 1/2		Cardiomyocytes
Mitochondrial biogenesis (Transcriptional- regulation)	TFAM	Transcription Factor A, Mitochondrial		Cardiomyocytes
